# Comprehensive Analysis of Aldehyde Dehydrogenases (ALDHs) and Its Significant Role in Hepatocellular Carcinoma

**DOI:** 10.1007/s10528-021-10178-0

**Published:** 2021-12-20

**Authors:** Senbang Yao, Wenjun Chen, He Zuo, Ziran Bi, Xiuqing Zhang, Lulian Pang, Yanyan Jing, Xiangxiang Yin, Huaidong Cheng

**Affiliations:** 1 Department of Oncology, The Second Affiliated Hospital of Anhui Medical University, No. 678 Furong Road, Hefei, 230601 Anhui Province China; 2 Department of Oncology, Anhui Medical University, No. 81 Meishan Road, Hefei, 230032 Anhui Province China

**Keywords:** HCC, ALDHs, Biomarkers, Integrated bioinformatics analysis

## Abstract

Oxidative DNA damage is closely related to the occurrence and progression of cancer. Oxidative stress plays an important role in alcohol-induced hepatocellular carcinoma (HCC). Aldehyde dehydrogenase (ALDH) is a family of enzymes that plays an essential role in the reducing oxidative damage. However, how ALDHs family affects alcohol-related HCC remains obscure. We aimed to explore the correlation between the differential expression of ALDHs in patients with HCC and pathological features, as well as the relationship between ALDHs and prognosis, and finally analyze the possible mechanism of ALDHs in targeted therapy of HCC. The data of HCC were downloaded from The Cancer Genome Atlas (TCGA) database. This research explored the expression and prognostic values of ALDHs in HCC using Oncomine, UALCAN, Human Protein Atlas, cBioPortal, Kaplan–Meier plotter, GeneMANIA, Tumor Immune Estimation Resource, GEPIA databases, and WebGestalt. Low mRNA and protein expressions of ALDHs were found to be significantly associated with tumor grade and clinical cancer stages in HCC patients. In particular, the loss of ALDH expression is more obvious in Asians, and its effect on prognosis is far more significant than that in the White race. Our findings play an important role in the study of prognostic markers and anti-liver cancer therapeutic targets for the members of the ALDHs family, especially in patients with liver cancer in Asia.

**Supplementary Information:**

The online version contains supplementary material available at 10.1007/s10528-021-10178-0.

## Introduction

Hepatocellular carcinoma (HCC) is a malignant tumor that can extremely affect human health. The most common reason for HCC includes hepatitis virus and alcohol drinking (Siegel et al. 2021). HCC is the sixth most common reason of cancer mortality in female patients and the second leading reason of cancer mortality in male patients (Zhu et al. 2015). As an area with high incidence of liver cancer, the prevention and treatment of HCC in China are facing a severe situation (Zhang et al. 2020). Despite remarkable advances in the progress for HCC, the overall survival and progression-free survival of HCC patients remain pessimistic. Early detection and treatment of HCC are very important to improve the prognosis of patients with HCC, so it is important to explore the predictive indexes related to the progress and prognosis of HCC (Song et al. 2020; Zeng et al. 2021). However, up to now, the biomarkers used to predict the prognosis of liver cancer are still very limited, which is extremely disadvantageous to patients in areas with high incidence of liver cancer such as Asia.

A family of detoxifying enzymes called acetaldehyde dehydrogenase (ALDHs) has been a hot topic in toxicology and cancer biology because its role in detoxifying aldehydes accumulated through metabolism has been elucidated and we are exposed to these aldehydes in the environment (Chen et al. 2014). The 19 genes of the ALDHs family play important roles in aldehyde detoxification, amino acid metabolism, embryogenesis and development, neurotransmission, oxidative stress, and cancer progression (Singh et al. 2013). Acetaldehyde is the main pathogenic factor of HCC, and there are abundant studies on the relationship between alcohol metabolic pathway and HCC. The aldehyde dehydrogenases (ALDHs) family has attracted wide attention as a key factor in ethanol metabolism (Wang et al. 2021). ALDHs play an important role in the progression of alcoholic liver disease (Seitz et al. 2018). Its role in liver fibrosis has also been explored by many scholars (Gao et al. 2018). However, there are few reports on the relationship between ALDHs and HCC.

In this research, we explored this problem by identifying the transcriptional and protein expression patterns of the ALDHs family through The Cancer Genome Atlas (TCGA), Oncomine, and Human Protein Atlas (HPA) databases. Then we used multi-dimensional analysis, evaluated functional networks and genomic alterations related to ALDHs in HCC, and explored its effect in tumor immunity. In addition, we also analyzed the clinical features and prognostic value of ALDHs family members in HCC. Our study shows the biological function and prognostic value of ALDHs in HCC, which will be beneficial to the diagnosis and treatment of HCC.

## Materials and Methods

### Differential Expression of ALDHs at Transcriptional Level

The Cancer Genome Atlas (TCGA) is a well-known cancer genomics project. It collects genomic information of more than 20,000 primary cancers and matches 33 normal samples of cancer types (Tomczak et al. 2015). The Gene Expression Profiling Interactive Analysis (GEPIA) is a database that includes 9736 tumors and 8587 normal samples from TCGA and the GTEx projects. We used GEPIA to explore the difference of ALDHs expression between cancer tissues and corresponding normal tissues in TCGA data (Tang et al. 2017). UALCAN is an extensive, usable web resource for analyzing cancer genomic information. It provides easy access to publicly available oncology data, such as TCGA, MET500, and CPTAC (Chandrashekar et al. 2017). We used UALCAN to explore the different tumor stages, tumor grades, or other clinicopathological features of ALDHs in HCC and classified them into different tumor subgroups.

Oncomine was born in October 2003, with 40 microarray data sets and nearly 100 differential expression analyses, allowing users to query differential expression results for a gene of interest across collected data sets (Rhodes et al. 2007). We analyzed the mRNA expression of ALDHs family members in HCC tissues and their adjacent normal control samples by Oncomine database. The specific parameters are as follows: *p*-value < 0.001, gene rank = 10%, fold change = 2.

### Differential Expression of ALDHs at Protein Level

In order to study the expression of ALDHs in HCC at the protein level, the HPA was used to directly observe the immunohistochemical images of ALDHs family proteins in normal and HCC specimens (Thul et al. 2017). The protein expression of ALDHs in normal liver tissue exists in the tissue module, which contains 44 kinds of normal human tissue protein expression data derived from the antibody-based protein profiling using immunohistochemistry.

### Functional Clustering and Molecular Network Construction of ALDHs in HCC

GeneMANIA is a web interface for generating hypotheses about gene function, analyzing gene lists, and prioritizing genes for functional assays (Warde-Farley et al. 2010). In our study, we submitted members of the ALDHs family to GeneMANIA to clarify the functional association network between ALDHs and its related genes. Specific network categories include shared protein domains, co-expression, physical interactions, predicted, co-localization, and genetic interactions.

WebGestalt (WEB-based Gene SeT AnaLysis Toolkit) is a functional enrichment analysis tool. GO functions and pathways of ALDHs and their related genes were enriched by WebGestalt. The GO functional enrichment was performed in the biological process no Redundant (BP), cellular component no Redundant (CC), and molecular function no Redundant (MF), and the KEGG pathway was performed by pathway analysis.

### Tumor Immunology Analysis

Tumor immunology was estimated using TIMER (Tumor Immune Estimation Resource) (Li et al. 2017), which is a web server for comprehensive analysis of tumor-infiltrating immune cells. The immune infiltration estimation of ALDHs was performed in LIHC (liver hepatocellular carcinoma) by TIMER. We explored ALDHs expression in LIHC and the correlation of ALDHs expression with the abundance of immune infiltrates, including B cells, CD4+T cells, CD8+T cells, neutrophils, macrophages, and dendritic cells, as well as the tumor purity. And we showed the purity-corrected partial Spearman’s rho value and statistical significance by drawing the scatter plots of ALDHs.

### Mutation and Survival Analysis of ALDHs in LIHC

The cBioPortal is an open network platform based on TCGA database that integrates data mining, data integration, and visualization (Cerami et al. 2012). An overview of genetic alterations per sample in ALDHs was displayed in OncoPrint. It was used to analyze ALDHs alterations in the TCGA LIHC patients. The search parameters included mutation, CNVs, mRNA expression, and survival.

In this research, the prognostic value of mRNA expression of ALDHs in HCC was analyzed by The Kaplan–Meier plotter, which is used to assess the effect of 54 k genes (mRNA, miRNA, and protein) on survival in 21 cancer types. Sources from the databases include GEO, EGA, and TCGA. The two patient cohorts are compared by a Kaplan–Meier survival plot, and the hazard ratio with 95% confidence intervals and log-rank P value are calculated. Databases and clinical data are supervised and extended regularly (Nagy et al. 2021).

### Statistical Analysis

The expression of ALDHs was analyzed by using independent samples *t*-test or a paired sample Student’s *t*-test as appropriate. Kaplan–Meier curves were used to compare the survival time differences. The log-rank test *p* < 0.05 indicates the significance of survival time differences. We used Wilcoxon signed-rank test, one-way ANOVA test, and logistic regression to explore the relationships between clinicopathologic features and the expression of ALDHs. The correlation between ALDHs and immune signature score or gene expression levels was calculated by using the Spearman method. Statistical analysis and plots were produced by R (v.3.5.1).

## Results

### Basic Information of ALDHs

**Fig. 1:**
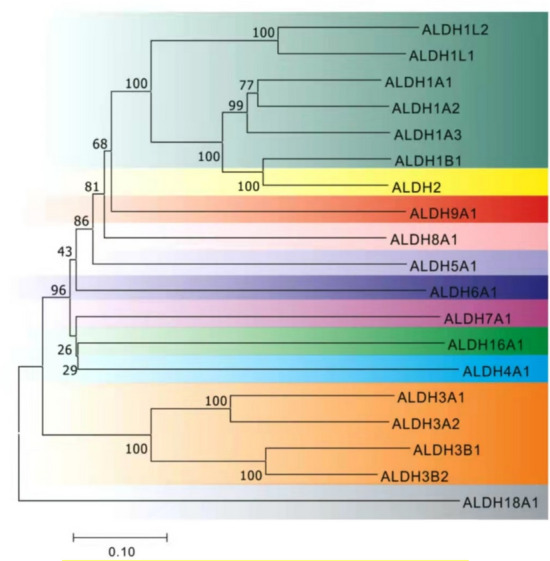
The phylogenetic tree of ALDHs family

In order to have a full grasp of the ALDHs family gene function, the substrates and products of ALDHs family were summarized (Table 1). Literature resources are as follows: ALDH4A1 (Pemberton and Tanner 2013), ALDH7A1 (Bok et al. 2007), ALDH1A1 (Verma et al. 2021), ALDH1A2 (Verma et al. 2021), ALDH1A3 (Verma et al. 2021), ALDH2 (Amanuma et al. 2015), ALDH1L1 (Krupenko et al. 2019), ALDH1B1 (Stagos et al. 2010), ALDH3B2 (Yin 2017), ALDH8A1 (Singh et al. 2015), ALDH6A1 (Kedishvili et al. 1992), ALDH16A1 (Vasiliou et al. 2013), ALDH3A1, ALDH3A2, ALDH3B1, ALDH5A1, ALDH7A1, ALDH9A1, ALDH18A1 (Koppaka et al. 2012). In addition, the phylogenetic tree of ALDHs family was structured (Fig. 1). Since several members of the ALDHs family are involved in the metabolism of different aldehydes. The results in Table 1 imply functional redundancy among the ALDHs family. Part of the downregulated ALDHs gene function may be accomplished by normally expressed genes. The evolutionary distance of ALDHs gene family members was demonstrated in the phylogenetic tree. It can be seen from Table 1 that ALDH2 and ALDH1A1, ALDH1A2, ALDH1A3, and ALDH1B1 have similar substrates and products. Interestingly, their evolutionary distances were relatively close (Fig. 1). But the expression of them was different. This expression difference may have a mutual regulatory effect with functional redundancy.

**Table 1:** The substrates and products of ALDHs family

Gene	Chromosome location	Preferred substrates	Products	References
ALDH1A1	9q21.13	Retinaldehyde Acetaldehyde	Retinoic acid (RA) Acetic acid	Verma et al. (2021)
ALDH1A2	15q22.1	Retinaldehyde Acetaldehyde	Retinoic acid (RA) Acetic acid	Verma et al. (2021)
ALDH1A3	15q26.3	Retinaldehyde	Retinoic acid (RA)	Verma et al. (2021)
ALDH1B1	9p11.1	Retinaldehyde Acetaldehyde	Retinoic acid (RA) Acetic acid	Stagos et al 2010)
ALDH1L1	3q21.2	10-formyltetrahydrofolate	Tetrahydrofolate (THF)	Krupenko et al. (2019)
ALDH1L2	2q23.3	10-formyltetrahydrofolate	Tetrahydrofolate (THF)	Krupenko et al. (2019)
ALDH2	12q24.2	Acetaldehyde	Acetic acid	Amanuma et al. (2015)
ALDH3A1	17p11.2	Aromatic Aliphatic Aldehydes	Aromatic acid Aliphatic acid	Koppaka et al. (2012)
ALDH3A2	17p11.1	Aliphatic Aldehydes	Aliphatic acid	Koppaka et al. (2012)
ALDH3B1	11q13.2	Octanal (octyl aldehyde)	Octylic acid	Koppaka et al. (2012)
ALDH3B2	11q13.2	Acetaldehyde	Acetic acid	Yin (2017)
ALDH4A1	1p36.13	Glutamate-1-semialdehyde	L-glutamate	Pemberton and Tanner (2013**)**
ALDH5A1	6p22.2	Succinate semialdehyde	Succinic acid	Koppaka et al (2012)
ALDH6A1	14q24.3	Malonate semialdehydes Methylmalonate semialdehydes	Acetyl-CoA Propionyl-CoA	Kedishvili et al. (1992)
ALDH7A1	5q31	α-aminoadipic semialdehyde	α-aminoadipate	Koppaka et al. (2012)
ALDH8A1	6q23.2	Retinaldehyde	Retinoic acid (RA)	S. Singh et al. (2015)
ALDH9A1	1q23.1	γ-aminobutyraldehyde	γ-aminobutyric acid (GABA)	Koppaka et al. (2012)
ALDH16A1	19q13.33	Non-catalytic function	Non-catalytic function	Vasiliou et al. (2013)
ALDH18A1	10q24.3	Glutamic-1-semialdehyde	L-glutamate	Koppaka et al. (2012)

### The Low mRNA Expression of Different ALDHs Family Members in Patients with HCC

**Fig. 2:**
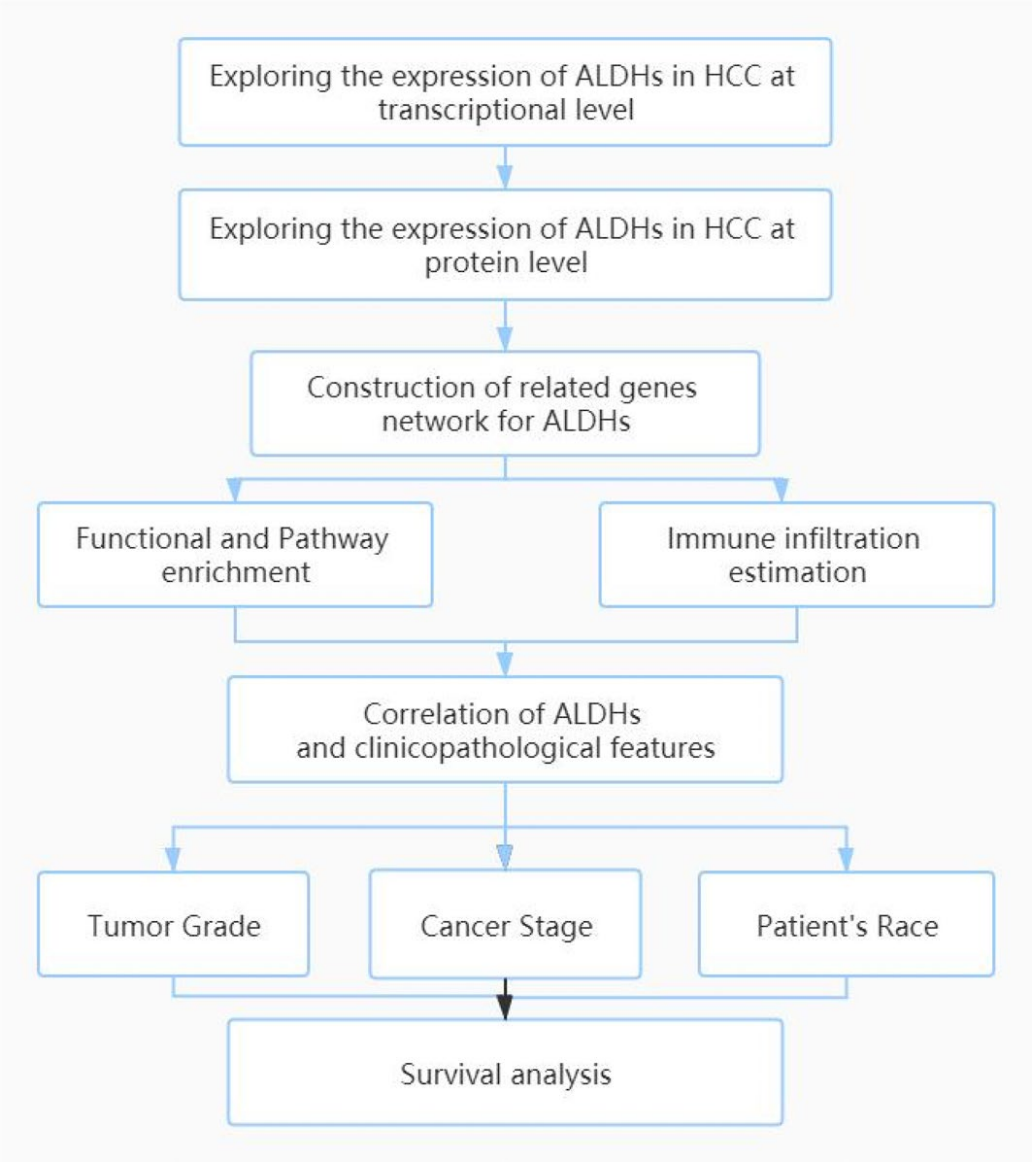
Design flow chart of the whole analysis process of this study

**Fig. 3:**
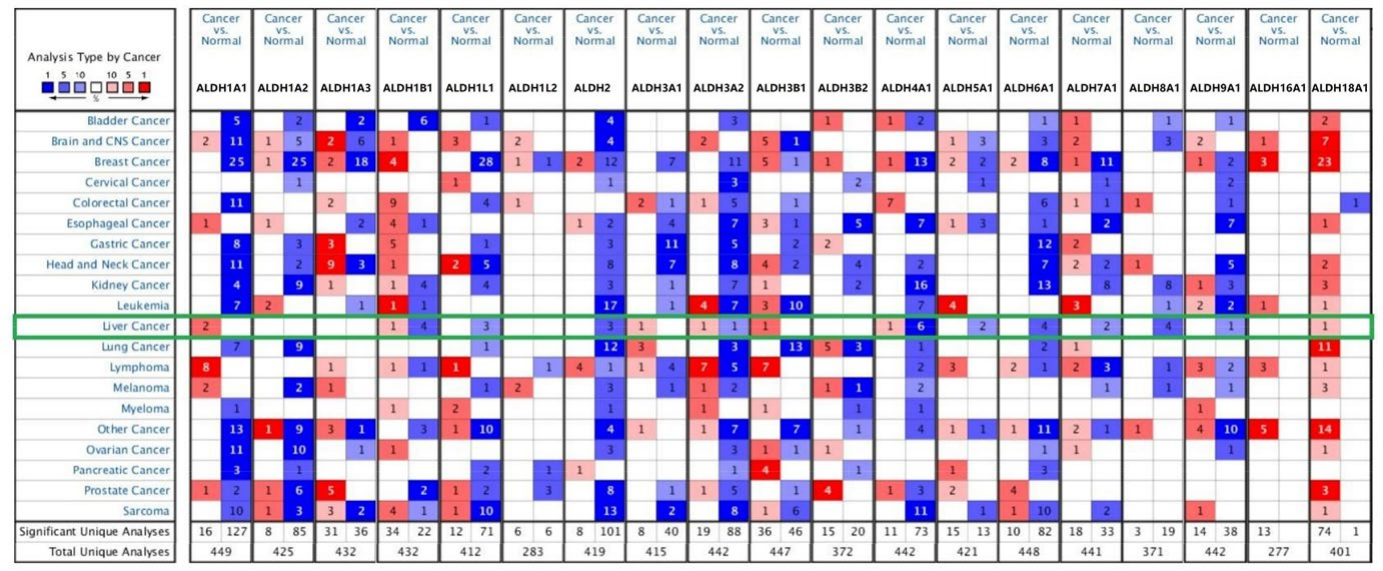
Transcriptional expressions of ALDHs family members in 20 types of cancers (Oncomine database). The data were compared by the *t*-test, cutoff *p*-value, and fold change as follows *p*-value < 0.001, gene rank = 10%, fold change = 2. Blue indicates downexpression, and red indicates overexpression (Color figure online)

The analysis process of this study is shown in the flow chart of Fig. 2. For the purpose of study the expression of ALDHs family members in patients with HCC, we used Oncomine database to analyze the expression differences between various cancers and corresponding normal tissues. The result shows that mRNA expressions of ALDH1B1, ALDH1L1, ALDH2, ALDH4A1, ALDH5A1, ALDH6A1, ALDH7A1, ALDH8A1, and ALDH9A1 were significantly lower in HCC tissues (Fig. 3). In the Roessler Liver 2 dataset, the mRNA expression of ALDH1B1, ALDH2, ALDH5A1, ALDH6A1, ALDH7A1, and ALDH9A1 was lower in HCC tissues compared with normal tissues with fold changes of 2.714, 2.185, 1.582, 3.481, 1.651, and 1.520 (*p* = 4.66E-52, 5.01E-61, 6.52E-21, 7.34E-57, 1.09E-18, 2.81E-29), respectively. Chen found a 2.638-fold decrease in mRNA expression of ALDH1B1 in HCC tissues. Mas and Wurmbach observed significant downexpression in ALDH4A1 mRNA in HCC tissues. Downregulation of mRNA expression of ALDH7A1 was also found in HCC tissues. Wurmbach also found that mRNA expression of ALDH8A1 in HCC was downexpression compared to normal tissues (Table 2).

**Table 2:** Transcription expression of ALDHs family members between HCC and normal liver tissues (Oncomine)

	Types of HCC VS liver	Fold change	*p*-value	*t*-test	References
ALDH1B1					
	Hepatocellular Carcinoma	− 2.714	4.66E− 52	− 17.357	Roessler Liver 2
	Hepatocellular Carcinoma	− 2.638	1.61E− 16	− 9.061	Chen Liver
ALDH1L1					
	Hepatocellular Carcinoma	− 5.249	3.25E− 6	− 5.682	Roessler Liver
	Hepatocellular Carcinoma	− 2.835	6.62E− 10	− 6.442	Chen Liver
ALDH2					
	Hepatocellular Carcinoma	− 2.815	5.01E− 61	− 20.816	Roessler Liver 2
	Hepatocellular Carcinoma	− 2.378	2.69E− 5	− 5.132	Wurmbach Liver
ALDH4A1					
	Hepatocellular Carcinoma	− 1.681	2.57E− 7	− 5.719	Mas Liver
	Hepatocellular Carcinoma	− 1.714	1.40E− 5	− 5.066	Wurmbach Liver
ALDH5A1					
	Hepatocellular Carcinoma	− 1.507	1.28E− 5	− 4.750	Roessler Liver
	Hepatocellular Carcinoma	− 1.582	6.52E− 21	− 9.857	Roessler Liver 2
ALDH6A1					
	Hepatocellular Carcinoma	− 3.053	2.52E− 21	− 11.013	Chen Liver
	Hepatocellular Carcinoma	− 3.481	7.34E− 57	− 19.760	Roessler Liver 2
ALDH7A1					
	Hepatocellular Carcinoma	− 1.935	7.58E− 6	− 5.026	Roessler Liver
	Hepatocellular Carcinoma	− 1.651	1.09E− 18	− 9.256	Roessler Liver 2
ALDH8A1					
	Hepatocellular Carcinoma	− 3.861	6.28E− 20	− 10.357	Chen Liver
	Hepatocellular Carcinoma	− 4.448	7.24E− 8	− 6.355	Wurmbach Liver
ALDH9A1					
	Hepatocellular Carcinoma	− 1.520	2.81E− 29	− 12.186	Roessler Liver 2

**Fig. 4:**
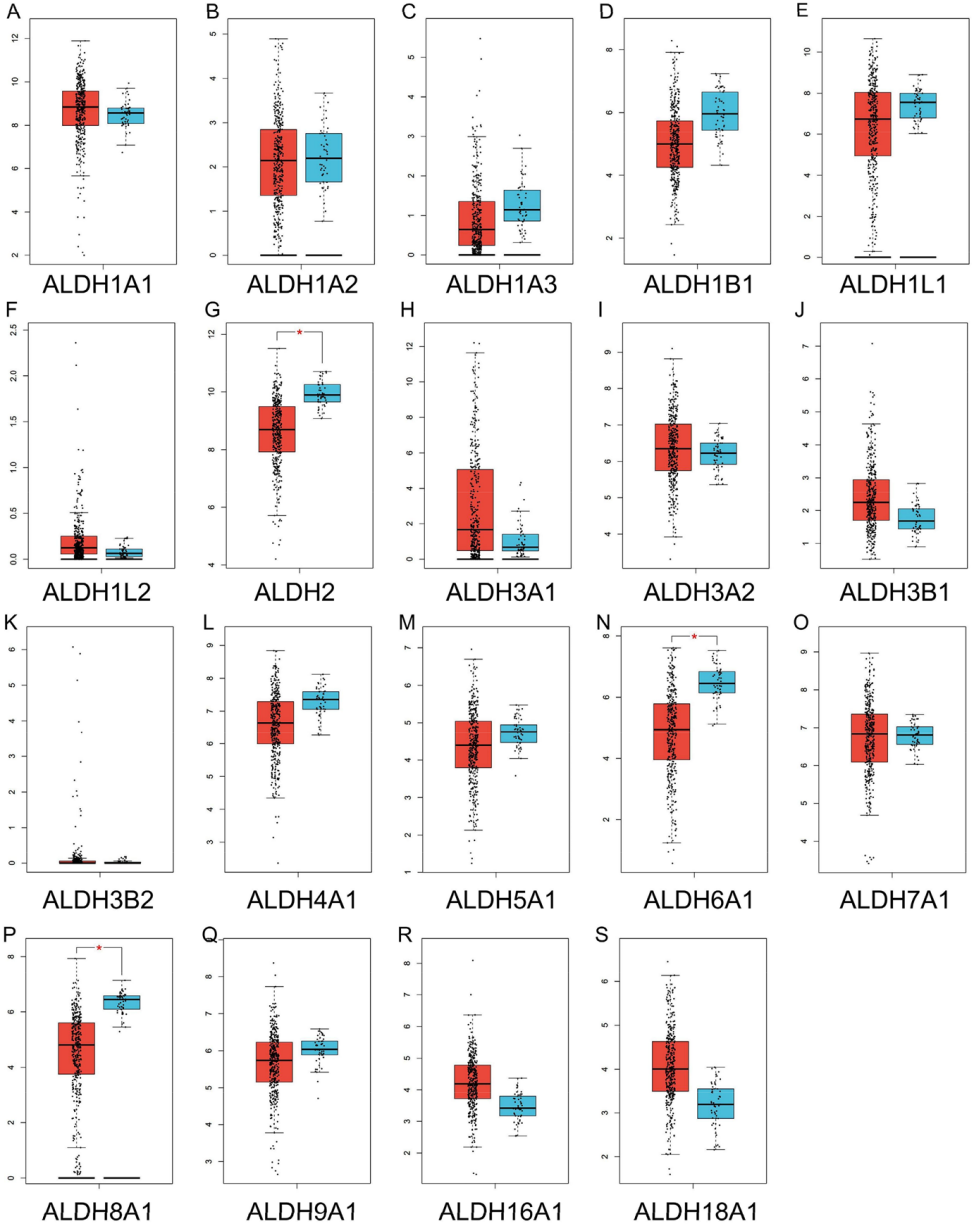
Low mRNA expressions of ALDHs family members in patients with HCC and normal liver tissues (TCGA database). The mRNA expressions of most ALDHs family members were significantly downregulated in patients with HCC from the TCGA database (A-S). ***p < 0.001

Then we further explored the mRNA expression of ALDHs family members through the TCGA database. The mRNA expression of most ALDHs members was downregulated in HCC tissues compared with normal samples, and there were significant differences in ALDH2, ALDH6A1 and ALDH8A1 among these groups, which was similar to the results of Oncomine analysis (Fig. 4).

Roessler Liver means the liver cancer-related microarray dataset uploaded by Roessler based on Human Genome U133A 2.0 Array platform. Roessler Liver 2 means the second liver cancer-related microarray dataset uploaded by Roessler based on Affymetrix Human Genome HT U133A Array platform. Chen Liver means the liver cancer-related microarray dataset uploaded by Chen based on Affymetrix Human Genome HT U133A Array platform.

### Difference of Protein Expression of ALDHs Family Members in Patients with HCC

**Fig. 5:**
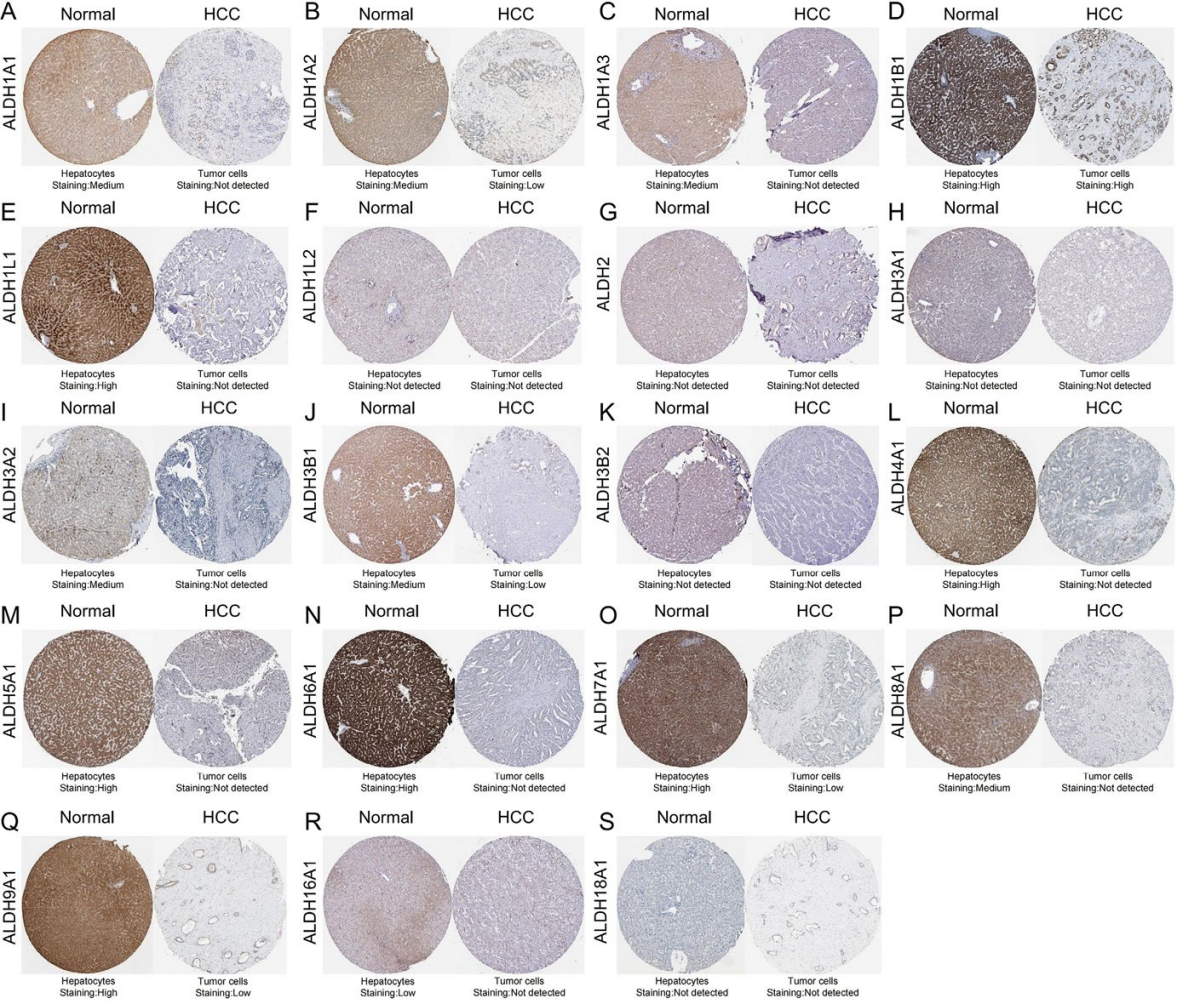
Immunohistochemistry images of different ALDHs in HCC tissues and normal liver tissues (HPA database)

We investigated the protein expression of ALDHs family members in HCC by HPA. Similar to the results of mRNA analysis, the expression of ALDHs protein was lower in HCC tissues detected by HPA (Fig. 5). Low protein expressions of ALDH1A1, ALDH1A2, ALDH1A3, ALDH1B1, ALDH1L1, ALDH3A2, ALDH3B1, ALDH3B2, ALDH4A1, ALDH5A1, ALDH6A1, ALDH7A1, ALDH8A1, ALDH9A1, and ALDH16A1 were found in HCC tissues, while their medium and high protein expressions were observed in normal liver tissues. Negative protein expressions of ALDH1L2, ALDH2, ALDH3A1, and ALDH18A1 were observed both in normal liver tissues and in HCC tissues (Fig. 5). Because HPA contains immunohistochemical results of liver cancer and normal tissue from different patients, then we quantified the stainings from different patients. The results showed that ALDH1A1, ALDH1L1, ALDH1L2, ALDH3A1, ALDH3A2, ALDH5A1, and ALDH8A1 gene expressions were significantly different between liver cancer and normal tissues (Additional file 1). Overall, our results showed that the expression of ALDHs family members was lower in patients with HCC, and ALDH2, ALDH6A1, and ALDH18A1 are significantly downregulated in HCC patients.

On the whole, the above results showed that the transcriptional and protein expression levels of ALDHs in HCC were low.

### Functional Enrichment Analysis of ALDHs Family Members in HCC

**Fig. 6:**
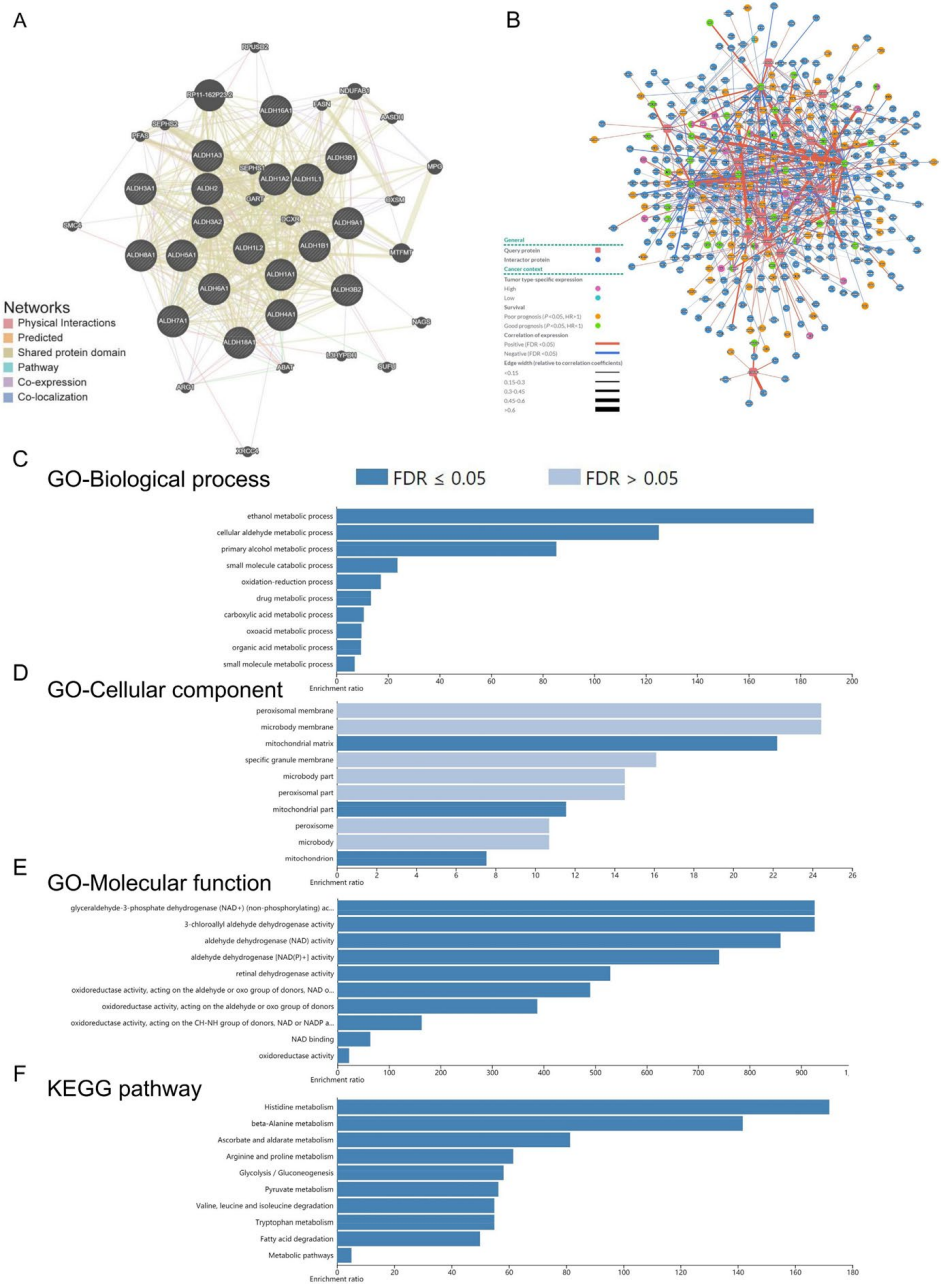
Function enrichment of ALDHs family members in HCC. **A** Network of ALDHs family members and their related genes was analyzed by GeneMANIA. **B** Interaction network analysis of ALDHs family members from PINA platform; **C** Cellular component; **D** Biological processes; **E** Molecular functions; **F** KEGG pathway analysis

The network of ALDHs family members and their related genes was constructed by PINA (Fig. 6A) and GeneMANIA (Fig. 6B). Through the functional network diagram, we can grasp the information of positive and negative related genes that interact with the ALDHs family, and the specific information is indicated in the diagram.

We used WebGestalt to analyze the GO function and pathway of ALDHs and its related genes.

Benjamini and Hochberg method was used for multiple-testing correction p-value, which also means FDR (false discovery rate). FDR reflects the probability of false positive rate in the test. By default, FDR < 0.05 was established at the statistically significant level. The biological processes such as ethanol metabolic process, cellular aldehyde metabolic process, and primary alcohol metabolic process were remarkably regulated by the ALDHs in HCC (Fig. 6C). Cellular components, including mitochondrial matrix, mitochondrial part, and mitochondrion (Fig. 6D). Besides, ALDHs also prominently affected the molecular functions (Fig. 6E), such as glyceraldehyde-3-phosphate dehydrogenase (NAD +) (non-phosphorylating) activity, 3-chloroallyl aldehyde dehydrogenase activity, and aldehyde dehydrogenase (NAD) activity.

Through KEGG analysis, we found that the pathways involved in ALDHs include histidine metabolism, beta-alanine metabolism, ascorbate and aldarate metabolism, and glycolysis/ gluconeogenesis (Fig. 6F).

### Analysis of Correlation Between mRNA Expression of ALDHs Family Members and HCC Immune Infiltration Level

**Fig. 7:**
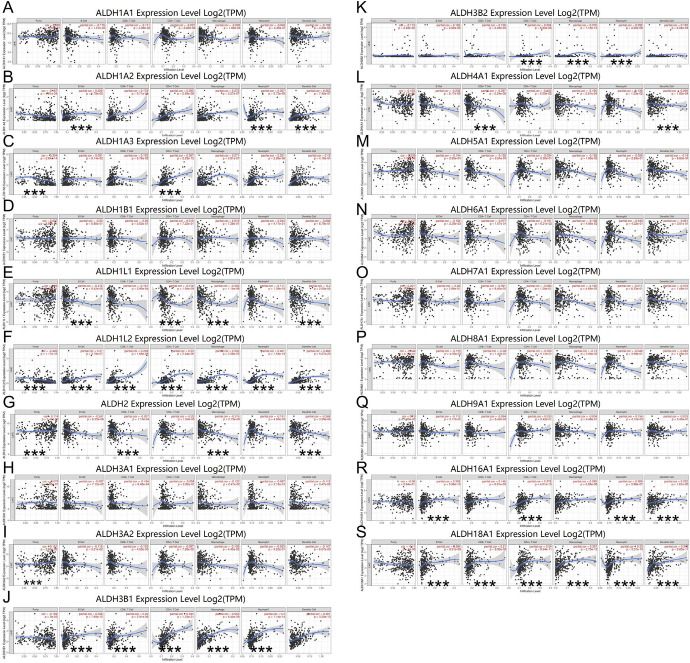
Association of mRNA expression of ALDHs family members with immune infiltration level in HCC. The mRNA expression of ALDHs family members was significantly related to the immune infiltration level in HCC (**A**–**S**)

TIMER database was used to explore the relationship between ALDHs family members and HCC immune infiltration. The results showed that the mRNA expressions of ALDH1A3, ALDH1L2, ALDH2, and ALDH3A2 were obviously related to tumor purity. The correlation of mRNA expression of ALDH1A2, ALDH1L1, ALDH1L2, ALDH3B1, ALDH16A1, and ALDH18A1 with B cell was statistically significant, while mRNA expression of ALDH1L2, ALDH2, ALDH3B1, ALDH4A1, and ALDH18A1 was obviously related to CD8+T cell. In addition, mRNA expression of ALDH1A3, ALDH1L1, ALDH1L2, ALDH3B1, ALDH3B2, ALDH16A1, and ALDH18A1 had significant correlations with infiltrating levels of CD4+T cells in HCC. The mRNA expressions of ALDH1L1, ALDH1L2, ALDH2, ALDH3B1, ALDH3B2, and ALDH18A1 were obviously related to macrophage. Since the M1-M2 macrophage polarization system was widely used in macrophage research. The correlation between subpopulation of macrophages and these genes was further explored (Additional file 2). Interestingly, the results showed that ALDH1L1 was negatively correlated with M1 macrophages and M2 macrophages. As we know, M1 macrophages have tumoricidal function, but M2 macrophages have been linked to tumor progression in tumor biology (Gerner et al. 2018). Therefore, this result has certain enlightening significance for exploring the regulation mechanism of ALDH1L1 in HCC in future. The mRNA expressions of ALDH1A2, ALDH1L2, ALDH3B1, ALDH3B2, ALDH16A1, and ALDH18A1 were obviously related to neutrophil infiltration. The mRNA expressions of ALDH1A2, ALDH1L1, ALDH1L2, ALDH2, ALDH4A1, ALDH16A1, and ALDH18A1 were obviously related to dendritic cell infiltration (Fig. 7).

### Correlation Between mRNA and Protein Expression of ALDHs Family Members and Clinicopathological Features of HCC Patients

**Fig. 8:**
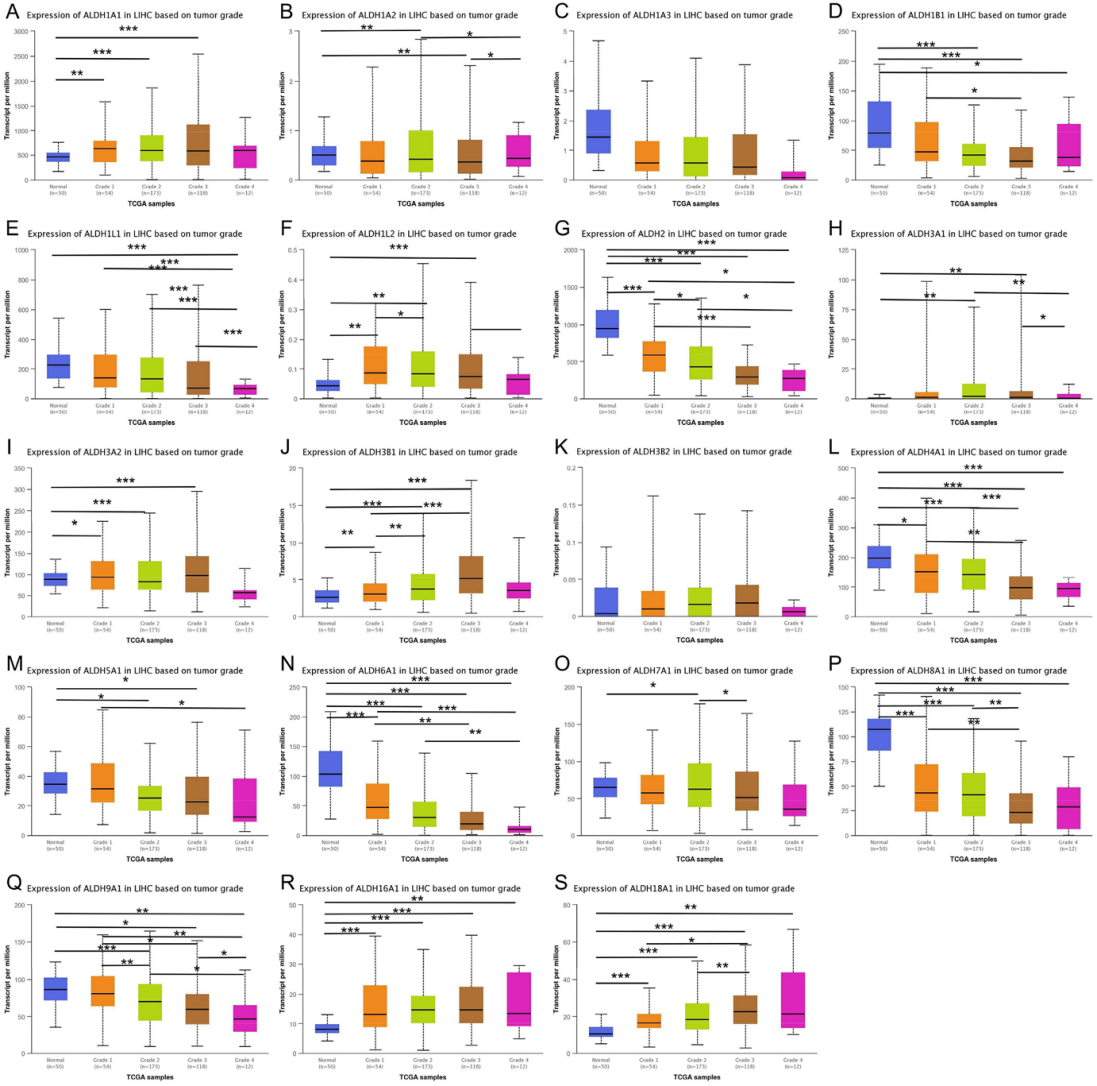
Association of mRNA expression of ALDHs family members with tumor grade of HCC patients. Boxplot showing the mRNA expression of ALDHs family members in normal individuals or in HCC patients in grades 1, 2, 3, or 4 (**A**–**S**). *p < 0.05; **p < 0.01; ***p < 0.001

**Fig. 9:**
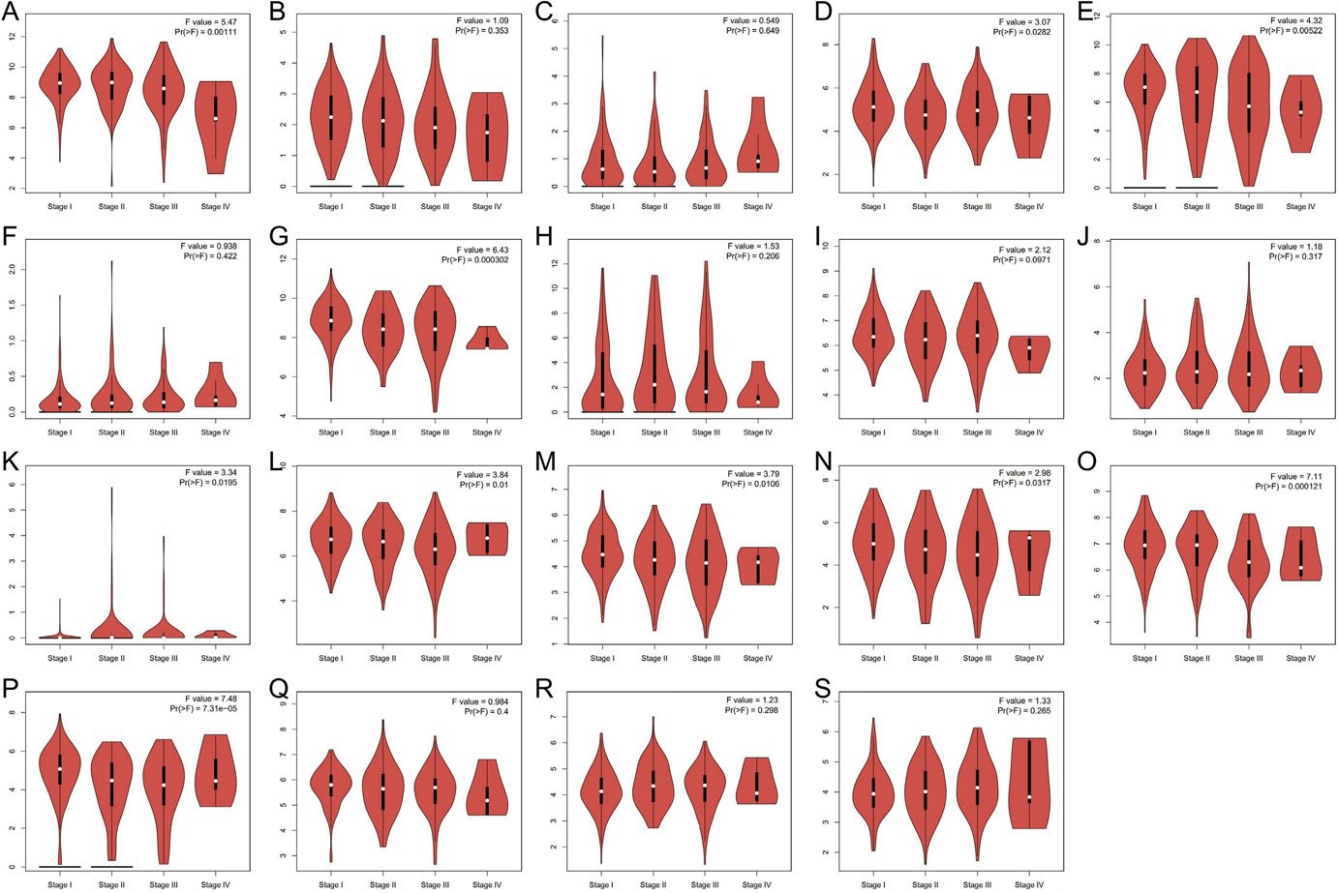
Association of mRNA expression of ALDHs family members with cancer stages of HCC patients. Violin plot showing the mRNA expression of ALDHs family members in HCC patients in cancer stages 1, 2, 3, or 4 (**A**–**S**). *p < 0.05; **p < 0.01; ***p < 0.001

We downloaded TCGA data through UALCAN, GEPIA, and Linkedomics and analyzed the relationship between mRNA expression of ALDHs family members and clinicopathological parameters (including tumor pathological grade, individual stage, and race) in patients with HCC. The mRNA expression level of ALDHs family members was significantly correlated with cancer stage. The more advanced the tumor stage, the lower the mRNA expression level of ALDHs. Similarly, the higher the malignant degree of the tumor grade, the lower the mRNA expression level of ALDHs (Figs. 8–9).

**Fig. 10:**
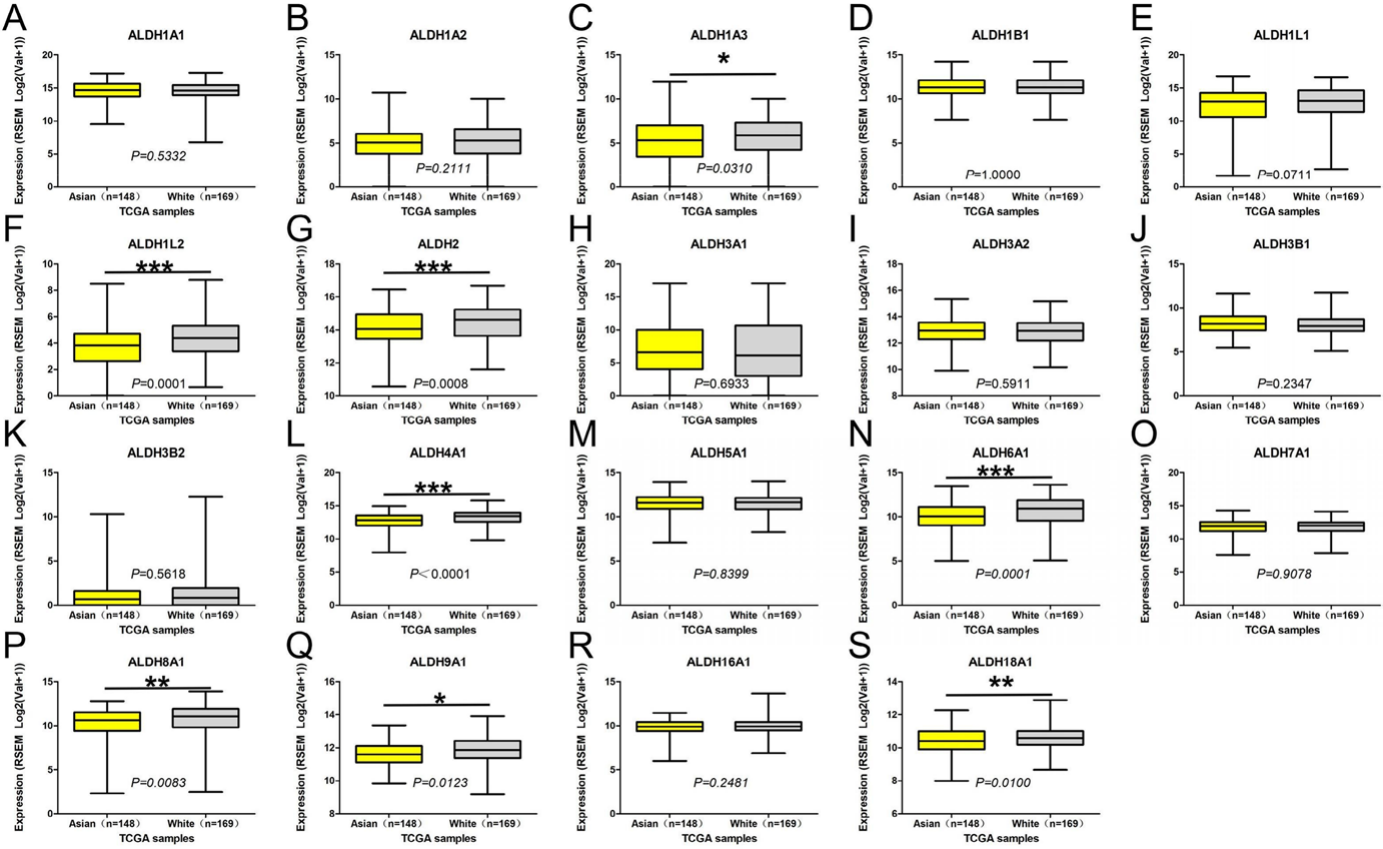
Association of mRNA expression of ALDHs family members with race of HCC patients. Boxplot showing the mRNA expression of ALDHs family members in Asian and the White race (**A**–**S**). *p < 0.05; **p < 0.01; ***p < 0.001

Then, we analyzed the relationship between race and mRNA expression level of ALDHs family members. The expression of ALDHs family members in Asian is lower than that in White as a whole, and the expression of ALDH1A3, ALDH1L2, ALDH2, ALDH4A1, ALDH6A1, ALDH8A1, ALDH9A1, and ALDH18A1 in Asian is significantly lower than that in White (Fig. 10).

In general, the expression of mRNA in part members of ALDHs was correlated with the clinicopathological parameters of patients with hepatocellular carcinoma.

### Mutation of ALDHs Family Members in Hepatocellular Carcinoma and Its Effect on Prognosis

**Fig. 11:**
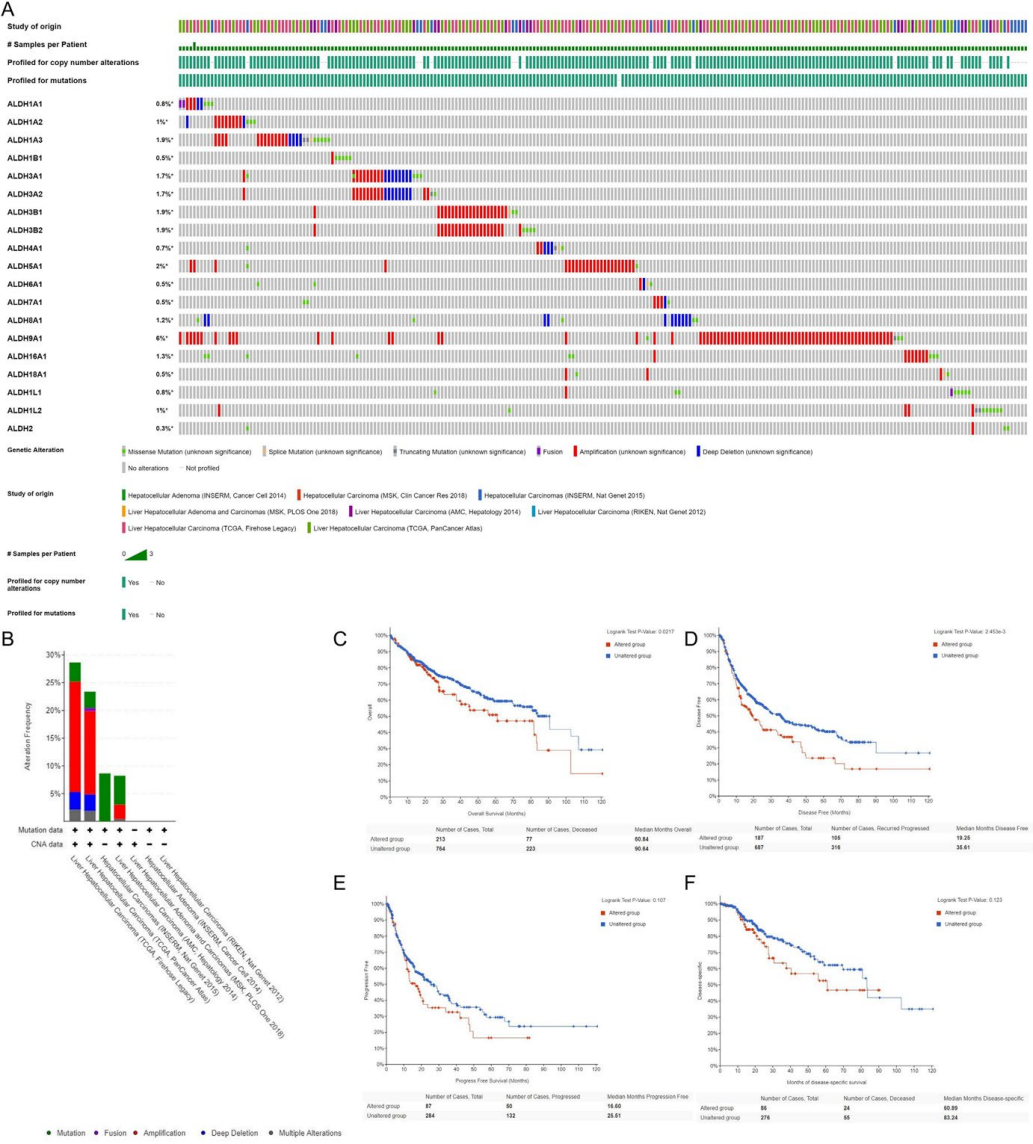
The frequency and type of ALDHs family mutations in 8 data sets of HCC (cBioPortal). (**A** An overview of ALDHs mutations. **B** Alteration frequency of ALDHs; **C** The overall survival of ALDHs mutation group was significantly shortened; **D** The disease-free survival of ALDHs mutation group was significantly shortened; **E** The progress-free survival of ALDHs mutation group was significantly shortened; **F** The disease-specific survival of ALDHs mutation group was significantly shortened

As shown in Fig. 11A, we explored the frequency and type of ALDHs family mutations in 8 data sets of hepatocellular carcinoma (containing 1507 samples) through cBioPortal. Alteration frequency in TCGA-Firehose Legacy, TCGA-PanCancer Atlas, INSERM-Nat Genet 2015, and AMC-Hepatology is 28%, 23%, 9%, and 8%, respectively (Fig. 10).

The mutation of ALDHs family members was significantly associated with the prognosis of HCC patients, and the OS, DFS, PFS, and DSS of ALDHs mutation group were significantly shortened.

From the overall survival analysis, there is a significant correlation between the mutation of ALDHs family and the poor prognosis of HCC patients. The normal expression of ALDHs family members is beneficial to HCC patients.

### Prognostic Value of mRNA Expression of ALDHs Family Members in Patients with HCC of Different Races

First, we used Kaplan–Meier plotter to analyze the relationship between mRNA expression of ALDHs family members and the prognosis of HCC patients. Lower mRNA expression of ALDHs was significantly associated with shorter OS and PFS of HCC patients (Table 3).

**Table 3:** Correlation of ALDHs mRNA expression and clinical prognosis in HCC by Kaplan–Meier plotter

ALDHs	Overall survival		Progression free survival	
	Hazard ratio	*p*-value	Hazard ratio	*p*-value
ALDH1A1	0.66 (0.46–0.95)	**0.0236**	0.71 (0.51–0.98)	**0.0353**
ALDH1A2	0.69 (0.48–0.97)	**0.0332**	0.85 (0.63–1.15)	0.3021
ALDH1A3	0.63 (0.44–0.90)	**0.0107**	0.67 (0.48–0.92)	**0.0136**
ALDH1B1	0.56 (0.36–0.87)	**0.0089**	0.89 (0.66–1.19)	0.4299
ALDH1L1	0.69 (0.49–0.98)	**0.0375**	0.66 (0.49–0.89)	**0.0065**
ALDH1L2	0.67 (0.47–0.95)	**0.0250**	0.73 (0.54–0.98)	**0.0379**
ALDH2	0.42 (0.29–0.60)	**1.3E-06**	0.53 (0.38–0.74)	**0.0001**
ALDH3A1	0.81 (0.56–1.16)	0.2475	1.31 (0.97–1.78)	0.0782
ALDH3A2	0.73 (0.50–1.06)	**0.0990**	0.73 (0.54–0.99)	**0.0410**
ALDH3B1	1.53 (1.08–2.18)	0.0170	1.17 (0.85–1.61)	0.3375
ALDH3B2	1.68 (1.14–2.48)	0.0076	1.26 (0.93–1.70)	0.1374
ALDH4A1	0.72 (0.51–1.02)	0.0632	0.53 (0.36–0.78)	**0.0009**
ALDH5A1	0.44 (0.31–0.62)	**2.2E-6**	0.63 (0.45–0.89)	**0.0084**
ALDH6A1	0.57 (0.40–0.81)	**0.0013**	0.69 (0.52–0.93)	**0.0150**
ALDH7A1	0.50 (0.35–0.71)	**7.7E-05**	0.66 (0.48–0.89)	**0.0072**
ALDH8A1	0.54 (0.35–0.84)	**0.0050**	0.63 (0.46–0.86)	**0.0032**
ALDH9A1	0.53 (0.36–0.78)	**0.0011**	0.61 (0.45–0.82)	**0.0012**
ALDH16A1	0.62 (0.42–0.90)	**0.0120**	0.67 (0.5–0.9)	**0.0075**
ALDH18A1	1.40 (0.97–2.01)	0.0740	1.39 (1.00–1.93)	0.0475

Statistically significant *p* values are shown in bold

Since the incidence of liver cancer in Asians is much higher than that of Whites, we conducted a subgroup analysis of the expression of ALDHs family in liver cancer patients of different races. And we were surprised to find that the difference in the expression of ALDHs family has a far greater impact on the prognosis of Asian liver cancer patients than Whites. The low expression of 15 genes in the ALDHs family is significantly associated with the poor prognosis of Asian liver cancer patients, while there are only 3 genes in White liver cancer patients (Fig. 11).

**Fig. 12:**
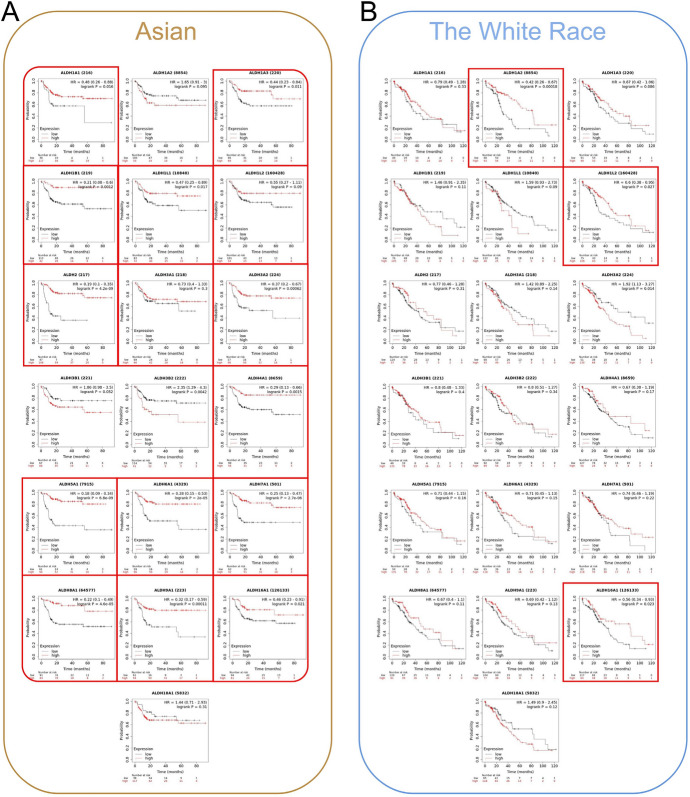
The effect of ALDHs expression on survival and prognosis in different races. **A** Correlation between the expression of ALDHs and the prognosis of HCC patients in Asia. **B** Correlation between the expression of ALDHs and the prognosis of HCC patients in the White race

These results suggest that differences in gene expression of ALDHs family of different races will affect the progression and prognosis of liver cancer (Fig. 12).

## Discussion

Alcohol and acetaldehyde are considered to be clear carcinogens; previous research described the features and mutational landscape of oxidative DNA damage caused by acetaldehyde, an endogenous and alcohol-derived metabolite (Garaycoechea et al. 2018; Vella et al. 2011). Aldehydes produced by alcohol metabolism are one of the major causes of HCC. Aldehyde dehydrogenases (ALDHs) play an important role in detoxifying aldehydes that accumulate through metabolism. On account of aldehyde dehydrogenases (ALDHs) that oxidize aldehydes to the corresponding carboxylic acids using either NAD or NADP as a coenzyme, thus the ALDHs family plays an important role in the metabolic degradation of carcinogens such as acetaldehyde (Muzio et al. 2012).

In fact, the expression and role of ALDHs family in cancer are receiving widespread attention. Currently, there are many studies to investigate the potential prognostic and diagnostic role of ALDHs. The prognostic significance of ALDH1A1 in breast cancer (Althobiti et al. 2020), ALDH1 in rectal cancer (Deng et al. 2014), ALDH1 in esophageal cancer (Hwang et al. 2014), and ALDH2 in oropharyngeal cancer (Shinomiya et al. 2017) has been explored by scholars.

As a member of ALDHs family, there are many studies on the relationship between ALDH2 and liver cancer. Asian drinkers have a higher risk of developing cancer because of mutations in ALDH2 and ALDH2 deficiency exacerbates alcohol-associated HCC development both in patients and in mouse models. One study found that ALDH2 deficiency had an increased risk of HCC development in cirrhotic HBV patients with alcohol drinking but not in those without alcohol drinking (Seo et al. 2019). However, other members of the ALDHs family have similar functions to ALDH2. Tomita et al. identified ALDH1 is a marker of tumor stem cells (CSC), where it is participating in self-renewal, differentiation, and self-protection (Tomita et al. 2016). Previous studies have shown that ALDH1A2 is a candidate tumor suppressor gene in ovarian cancer and enhancing ALDH1A2-linked signaling might provide new opportunities for therapeutic intervention (Choi et al. 2019). ALDH3A1 acts as a prognostic biomarker and inhibits the epithelial–mesenchymal transition of oral squamous cell carcinoma through IL-6/STAT3 signaling pathway (Qu et al. 2020). ALDH5A1 mRNA expression was downregulated in OC patients compared with that in normal tissues and a high mRNA level of ALDH5A1 was associated with improved overall survival (Tian et al. 2017). Recent studies underscored ALDH1L1 as a candidate tumor suppressor and potential marker of aggressive cancers (Krupenko and Krupenko 2018). In view of this, their functions in the prediction and progression of liver cancer should also be paid attention to and studied in depth.

Our study showed that mRNA expressions of ALDH1B1, ALDH1L1, ALDH2, ALDH4A1, ALDH6A1, ALDH7A1, ALDH8A1, and ALDH9A1 were significantly lower in HCC tissues compared to normal tissues from the Oncomine database. The mRNA expressions of majority ALDHs family members were downregulated in HCC tissues from the TCGA database. And there were significant differences in ALDH2, ALDH6A1, and ALDH8A1 among these groups. The important role of these three genes in HCC has also been confirmed by many scholars (Shin et al. 2020; Seo et al. 2019; Grinberg et al. 2014). ALDH2 has been proven to play a carcinogenic role in HCC by modulating the activity of the ALDH2-acetaldehyde-redox-AMPK axis (Hou et al. 2017). Besides, similar results were found by the HPA in protein expressions. With further research, we found that proteins, including RP11-162P23.2, NDUFAB1, AASDH, SEPHS1, SEPHS2, GART, and DCXR, interacted with ALDHs family members through PPI network analysis by GeneMANIA. Our results suggest that the functional consequence of ALDHs mainly includes ethanol metabolic process, cellular aldehyde metabolic process, and primary alcohol metabolic process. These findings are consistent with the molecular pathways implicated in HCC carcinogenesis. Subsequently, we analyzed the association of ALDHs family members's expression with clinicopathological factors of HCC patients. The result shows a tendency that the lower the expression of ALDHS family members, the worse the tumor stage and grade in HCC patients. And according to the results of data analysis, the expressions of ALDH1A3, ALDH1L2, ALDH2, ALDH4A1, ALDH6A1, ALDH8A1, ALDH9A1, and ALDH18A1 related to the race of patients.

In the survival analysis, we found that the expression of ALDHs has significant survival differences between the Asian and White population. The expression of ALDHs family genes has a significantly stronger effect on the prognosis of Asian liver cancer patients than White patients. Some researchers found that polymorphisms on ALDH2 had a significant indirect effect on HCC risk, mediated through alcohol drinking (Liu et al. 2016). By analyzing the researches, we suspect that this may be due to more liver cancers caused by hepatitis B virus in Asian liver cancer patients. The interaction between ALDHs and hepatitis B is also worth exploring.

This study preliminarily explored the expression of ALDHs at RNA and protein levels. There are still some shortcomings in the research. Although, on the whole, ALDHs showed consistent downregulation of RNA and protein levels, there are still some inconsistencies. This is because RNA analysis results were derived from TCGA, while protein analysis results were derived from HPA, which included different patients. In further exploration, we will collect samples of liver cancer patients by ourselves. The expression of same sample will be tested at RNA and protein levels to provide more convincing research conclusions.

## Conclusion

In conclusion, our study identified that downregulation of ALDHs members in HCC was common. Moreover, ALDHs were significantly associated with individual cancer stage, nodal metastasis status, and patient’s race. Furthermore, high expressions of ALDHs were significantly related to longer OS in HCC patients. And the performance of this benefit among Asian HCC patients is much higher than that of Whites. In an overall view, our findings play an important role in the study of prognostic markers and anti-liver cancer therapeutic targets for members of the ALDHs family, especially in patients with liver cancer in Asia.

## Supplementary Information

Below is the link to the electronic supplementary material.

Supplementary file1 (XLS 50 KB)

Supplementary file2 (JPG 686 KB)

## Data Availability

The source data of this study were derived from the public repositories, as indicated in the section of “Materials and Methods” of the manuscript.
