# Supplementary figures and images for: Comprehensive Analysis of Aldehyde Dehydrogenases (ALDHs) and Its Significant Role in Hepatocellular Carcinoma
Source: Biochem Genet. 2021 Dec 20;60(4):1274–97. doi: 10.1007/s10528-021-10178-0 (PMC9270301; doi:10.1007/s10528-021-10178-0)

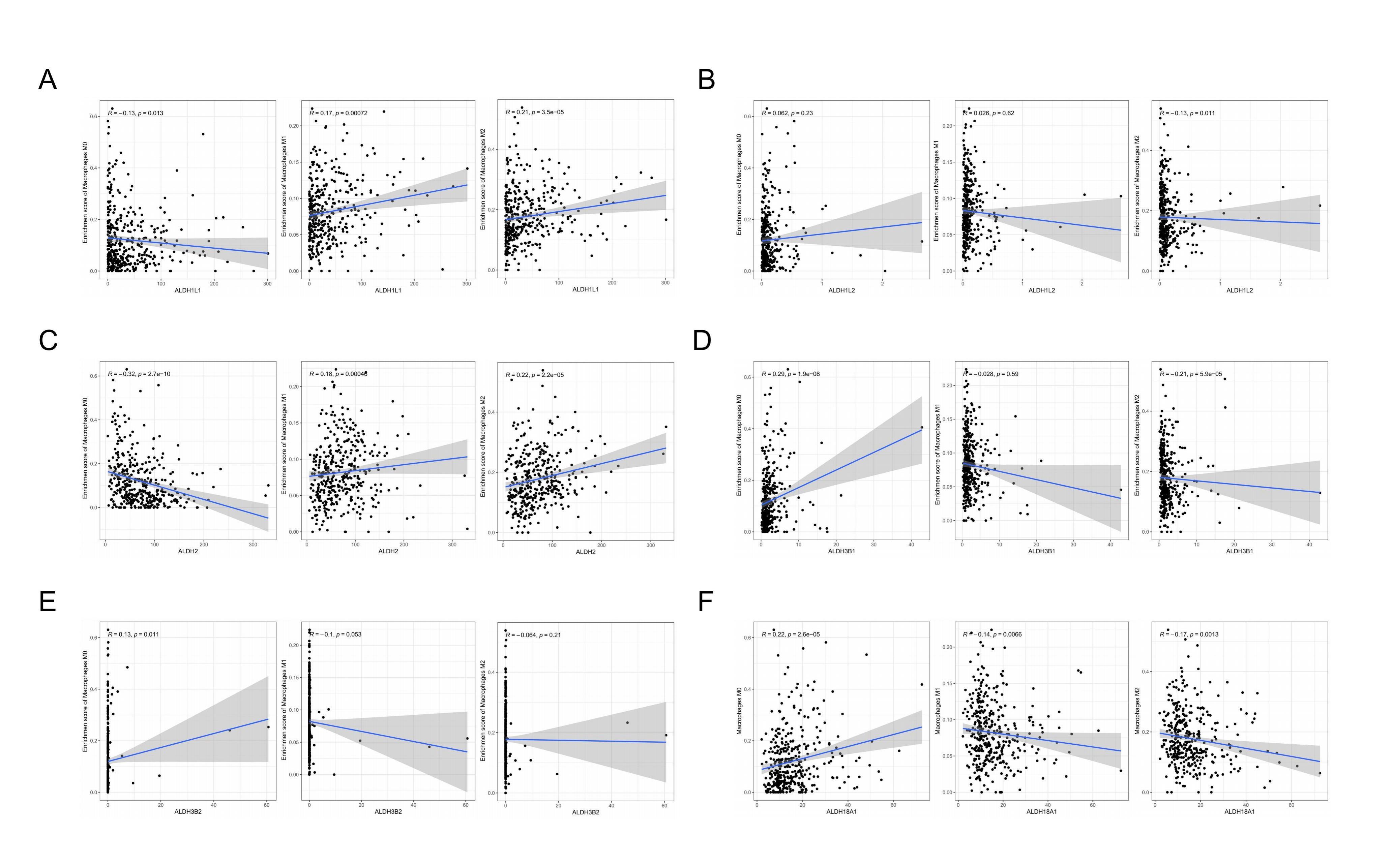

Supplement: Supplementary file 2 — Supplementary file2 (JPG 686 KB) [file 10528_2021_10178_MOESM2_ESM.jpg]
